# Indoor Air Quality and Health

**DOI:** 10.3390/ijerph14111286

**Published:** 2017-10-25

**Authors:** Alessandra Cincinelli, Tania Martellini

**Affiliations:** Department of Chemistry “Ugo Schiff”, University of Florence, via della Lastruccia 3, 50019 Sesto Fiorentino Florence, Italy; tania.martellini@unifi.it

In the last few decades, Indoor Air Quality (IAQ) has received increasing attention from the international scientific community, political institutions, and environmental governances for improving the comfort, health, and wellbeing of building occupants. Several studies on this topic have shown both qualitative and quantitative IAQ variations through the years, underlining an increase in pollutants and their levels. To this aim, IAQ-related standards and regulations, policies for non-industrial buildings, and monitoring plans have been developed in several countries. It has been estimated that people spend about 90% of their time in both private and public indoor environments, such as homes, gyms, schools, work places, transportation vehicles, etc.; thus, IAQ has a significant impact on health and quality of life in general. For many people, the health risks from exposure to indoor air pollution may be greater than those related to outdoor pollution. In particular, poor indoor air quality can be harmful to vulnerable groups such as children, young adults, the elderly, or those suffering chronic respiratory and/or cardiovascular diseases.

Indoor environments represent a mix of outdoor pollutants prevalently associated with vehicular traffic and industrial activities, which can enter by infiltrations and/or through natural and mechanical ventilation systems, as well as indoor contaminants, which originate inside the building, from combustion sources (such as burning fuels, coal, and wood; tobacco products; and candles), emissions from building materials and furnishings, central heating and cooling systems, humidification devices, moisture processes, electronic equipment, products for household cleaning, pets, and the behavior of building occupants (i.e., smoking, painting, etc.).

IAQ can be affected by various chemicals, including gases (i.e., carbon monoxide, ozone, radon), volatile organic compounds (VOCs), particulate matter (PM) and fibers, organic and inorganic contaminants, and biological particles such as bacteria, fungi, and pollen. The large number of variables that impact IAQ inevitably leads to a wide range of studies and scientific papers published in journals from many kinds of scientific subjects (e.g., chemistry, medicine, environmental sciences, etc.). To further underline the importance of IAQ studies, the present special issue was published. It includes 22 contributions by some of the main experts in the field of indoor air pollution in public and private buildings and related health concerns.

In particular, an indoor air sampling was monitored by Orecchio et al. [1] to determine 181 VOCs emitted from several sources (fuels, traffic, landfills, coffee roasting, a street-food laboratory, building work, indoor use of incense and candles, a dental laboratory, etc.) located in Palermo (Italy) by using canister auto-samplers and the gas chromatography-mass spectrometry technique for VOC analysis.

Concerning indoor air in residential houses, the study of Vilčeková et al. [2] attempted to provide more information about the IAQ of 25 houses in several cities of the Formal Yugoslav Republic of Macedonia. Air pollutants measured included humidity, total VOCs, PM, and sound pressure. The authors found interesting dependences between characteristics of buildings (year of construction, year of renovation, smoke, and heating system) and chemical-physical measurements (temperature, relative humidity, TVOC, PM_2.5_, and PM_10_) using statistical approaches (i.e., R software, Van der Waerden test).

The influence of particle size on human indoor exposure to airborne halogenated flame retardants (HFRs), released from consumer products, was investigated by La Guardia et al. [3]. Their findings demonstrated that the larger, inhalable air particulates carried the bulk (>92%) of HFRs and indicated that contributions and the bioavailability of respirable and inhalable airborne particles should both be considered in future risk assessment studies.

IAQ in enclosed environments was also studied by Chen et al. [4] who investigated the occurrence and levels of chemicals (including humidity, temperature, carbon monoxide, carbon dioxide, formaldehyde, TVOCs, ozone, PM_10_ and PM_2.5_, and microbial agent concentrations (i.e., bacteria and fungi) in North Taiwan underground subway stations).

Moreover, various studies have been conducted on the health risks of dampness and mold in houses, but few studies have been performed in workplaces and schools. The paper of Lanthier-Veilleux et al. [5] is an examination of the independent contribution of residential dampness or mold (i.e., visible mold, mold odor, dampness, or water leaks) to asthma, allergic rhinitis, and respiratory infections among students at the Université de Sherbrooke (Quebec, QC, Canada); while the work of Szulc et al. [6] evaluated the microbiological contamination at a plant biomass processing thermal power station located in Poland.

Among the factors that influence the estimation of human exposure to indoor air pollution, the pattern of human behavior and activity play a fundamental role. Odeh and Hussein [7] evaluated, for the first time, the human activity pattern of 285 subjects (17–63 years old) residents in Amman (Jordan) in order to use the outcomes in future human exposure studies.

Environmental tobacco smoke (ETS) is also considered a key contributor to indoor air pollution and public health. In comparison to the large body research on toxicological substances of ETS and concentrations of indoor ETS-dependent PM, less attention has been paid on the correlation between the odor concentration and the chemical composition of ETS. The odor concentrations of field ETS, second-hand smoke (SHS), and third-hand smoke (THS) in prepared samples were determined by Noguchi et al. [8] using the triangle-odor-bag method, while the chemical compositions of the same samples were determined by proton transfer mass spectrometry. Results of this study evidenced that the main contributing components to the odor of the field ETS samples (acetaldehyde, acetonitrile, acetic acid, and other unknown components with a mass-to-charge ratio (*m*/*z*) of 39 and 43) were different from those found in SHS and THS samples.

A potential threat to IAQ in indoor environments can be related to the contribution of outdoor pollutants concentrations and rates of infiltration, which affect the concentrations to which people are exposed indoors. Scheepers et al. [9] investigated the concentrations of volatile organic compounds (VOCs), acrolein, formaldehyde, nitrogen dioxide (NO_2_), respirable particulate matter (PM_4.0_ and PM_2.5_), and their respective benz(a)pyrene contents over a period of two weeks in indoor and outdoor locations at a university hospital, found that chemical IAQ was primarily driven by known indoor sources and activities, and did not show evidence of significant contributions of known outdoor local sources to any of the IAQ parameters measured.

In particular, the ventilation rate (VR) is a fundamental parameter affecting the IAQ and the energy consumption of buildings. The manuscript of Batterman [10] reviews the use of CO_2_ as a “natural” tracer gas for estimating VRs in school classrooms, and provides details and guidance for the steady-state, build-up, decay, and transient mass balance methods. The CO_2_ tracer approach was also used by Matthews et al. [11] within a large university building in Manchester to estimate air-exchange rates. The same authors presented an innovative approach based on the use of perfluorocarbon tracers to trace the amount of outdoor material penetrating into the university building and the flow of material within the building itself.

Minimizing indoor air pollutants is paramount to high performance schools, due to the potentially detrimental effects that VOCs, particulate matter including allergens and molds, and combustion gases may have on the health and wellbeing of students. In addition to their capacity to trigger asthma or allergy attacks, some of these pollutants are notorious for causing flu-like symptoms, headaches, nausea, and irritation of the eyes, nose, and throat. Moreover, a recent research suggests that a school’s physical environment also can play a major role in academic performance. However, newer designs, construction practices, and building materials for “green” buildings and the use of “environmentally friendly” products have the promise of lowering chemical exposure. Zhong et al. [12] determined VOC concentrations and IAQ parameters in 144 classrooms in 37 conventional and high performance elementary schools in the USA, and found that aromatics, alkanes, and terpenes were the most detected VOCs, whose concentrations did not show significant differences between the two kinds of schools.

This special issue also presents the relationships and potential conflicts between IAQ and passive houses and/or other highly energy-efficient buildings, focusing the attention on the influence of ventilation systems. Wallner et al. [13] investigated, between 2010 and 2012, whether occupants of two types of buildings (mechanical vs. natural ventilation) experience different health, wellbeing, and housing satisfaction outcomes, as well as whether associations with indoor air quality existed. The study evidenced that inhabitants of energy-efficient, mechanically ventilated homes rated the quality of indoor air and climate significantly higher and, independently of the type of ventilation, associations between vegetative symptoms (dizziness, nausea, headaches) and formaldehyde concentrations as well as between CO_2_ levels and perceived stale air were observed. 

More topics covered in this special issue are related to the IAQ in healthcare facilities together with the air cleanliness in operating theatres, which are fundamental aspects for preserving the health of both the patient and the medical staff. Numerous monitoring campaigns were performed by Romano et al. [14] to determine ultrafine particle concentrations in operating theatres equipped with upward displacement ventilation or with a downward unidirectional airflow system. The results demonstrated that the use of electrosurgical tools generate an increase of particle concentration in the surgical area as well as within the entire operating theatre area, strongly related to the surgical ventilation, ventilation principle, and electrosurgical tools used. Cipolla et al. [15] monitored the VOCs concentrations (including hydrocarbons, alcohols, and terpenes) using passive diffusive samplers in two different anatomical pathology wards in the same hospital, evidencing a different VOC contamination due to the structural difference of the buildings and different organizational systems.

Another theme that emerges from the studies presented in this special issue is the household air pollution (HAP) from the combustion of biomass fuels, including wood, agricultural residues, animal dung, coal, and charcoal, in open fires or traditional stoves. Such inefficient cooking and heating practices are still commonly used in developing countries and release many air pollutants, such as carbon monoxide, oxygenated organics, free radicals, and PM, in particular PM_2.5_, which may be linked to several health complications, including low birth weight, cardiovascular disease, tuberculosis, cataracts, and other respiratory complications.

The study of Kurti et al. [16] determined whether HAP exposure was associated with reduced lung function and respiratory and non-respiratory symptoms in Belizean adults and children, demonstrating that adults experienced greater respiratory and non-respiratory symptoms; whereas the research conducted by Medgyesi et al. [17] investigated the effects of exposure to biomass fuel cookstove emissions on women in rural Bangladesh, associated with acute elevated PM_2.5_ concentrations, and evidencing a decrease in pulmonary function. Novel evidence that using cleaner fuels such as liquefied petroleum gas (LPG) with respect to dirty fuels like wood/straw for domestic cooking is associated with a significant lower probability of chronic or acute diseases was demonstrated by Nie et al. [18], in their study on women in rural China. These findings support literature data showing that inefficient biomass burning stoves may cause high levels of HAP and threaten long-term health diseases. To reduce HAP in developing countries, clean cooking programs and strategic governmental policies should be adopted, taking into consideration the main factors influencing adoption beyond health, such as cost, taste, fears, and cultural traditions, as evidenced in the study of Hollada et al. [19] assessing the attitudes, preferences, and beliefs about traditional versus liquefied petroleum gas (LPG) stoves in primary cooks and their families in rural Puno, Peru.

Residential exposure to radon is strictly associated with lung cancer risk; thus, radon monitoring in households located in areas classified by United States–Environmental Protection Agency (US-EPA) as zones with high potential radon exposure is essential to safeguard the health of residents. Stauber et al. [20] presented a pilot study to monitor radon levels in 201 households located in Dekalb county (GA, USA), and found that radon exceeded EPA moderate risk levels in 18% of households and high risks in 4% of the homes tested, suggesting that a more extensive radon screening is needed in the entire county.

Taking into account the increasing IAQ concerns and complaints, it becomes important to develop a practical diagnostic tool for proper IAQ management. The study of Wong et al. [21], conducted in Hong Kong, proposes a stepwise IAQ screening protocol to facilitate cost-effective IAQ management among building owners and managers and to identify both lower and higher risk groups for unsatisfactory IAQ. Furthermore, the study of Marques and Pitarma [22] led to the development of an IAQ system through web access and mobile applications to monitor the IAQ of different building rooms in real time.

As seen, the contributions to this special issue cover a large area of IAQ-related studies, and it is expected that more deep research will be stimulated and conducted as a result of this special issue.
